# A girl with MIRAGE syndrome who developed steroid-resistant nephrotic syndrome: a case report

**DOI:** 10.1186/s12882-020-02011-4

**Published:** 2020-08-12

**Authors:** Sho Ishiwa, Koichi Kamei, Kanako Tanase-Nakao, Shinsuke Shibata, Kunihiro Matsunami, Ichiro Takeuchi, Mai Sato, Kenji Ishikura, Satoshi Narumi

**Affiliations:** 1 Division of Nephrology and Rheumatology, National Center for Child Health and Development, 2-10-1 Okura, Setagaya-ku, Tokyo, 157-8535 Japan; 2 Department of Pediatric Nephrology, Tokyo Women’s Medical University, Tokyo, Japan; 3 Department of Molecular Endocrinology, National Research Institute for Child Health and Development, Tokyo, Japan; 4 Electron Microscope Laboratory, Keio University School of Medicine, Tokyo, Japan; 5 Department of Pediatrics, Gifu Prefectural General Medical Center, Gifu, Japan; 6 Center for Pediatric Inflammatory Bowel Disease, Division of Gastroenterology, National Center for Child Health and Development, Tokyo, Japan; 7 Department of Pediatrics, Kitasato University School of Medicine, Sagamihara, Kanagawa Japan

**Keywords:** MIRAGE syndrome, *SAMD9*, Steroid-resistant nephrotic syndrome (SRNS), Focal segmental glomerulosclerosis (FSGS), Endocytosis

## Abstract

**Background:**

MIRAGE syndrome is a recently discovered rare genetic disease characterized by myelodysplasia (M), infection (I), growth restriction (R), adrenal hypoplasia (A), genital phenotypes (G), and enteropathy (E), caused by a gain-of-function mutation in the *SAMD9* gene. We encountered a girl with molecularly-confirmed MIRAGE syndrome who developed steroid-resistant nephrotic syndrome.

**Case presentation:**

She was born at 33 weeks gestational age with a birth weight of 1064 g. She showed growth failure, mild developmental delays, intractable enteropathy and recurrent pneumonia. She was diagnosed as MIRAGE syndrome by whole exome sequencing and a novel *SAMD9* variant (c.4615 T > A, p.Leu1539Ile) was identified at age four. Biopsied skin fibroblast cells showed changes in the endosome system that are characteristic of MIRAGE syndrome, supporting the genetic diagnosis. Proteinuria was noted at age one, following nephrotic syndrome at age five. A renal biopsy showed focal segmental glomerulosclerosis (FSGS) with immune deposits. Steroid treatment was ineffective. Because we speculated that her nephrosis was a result of genetic FSGS, we decided not to introduce immunosuppressive agents and instead started enalapril to reduce proteinuria. Although her proteinuria persisted, her renal function was normal at age eight.

**Conclusions:**

This is the first detailed report of a MIRAGE syndrome patient with nephrotic syndrome. Because patients with MIRAGE syndrome have structural abnormalities in the endosomal system, we speculate that dysfunction of endocytosis in podocytes might be a possible mechanism for proteinuria.

## Background

MIRAGE syndrome is a recently discovered rare genetic disease characterized by myelodysplasia (M), infection (I), growth restriction (R), adrenal hypoplasia (A), genital phenotypes (G), and enteropathy (E), caused by a gain-of-function mutation in the *SAMD9* gene on the arm of chromosome 7 (7q21.2) [1]. Although the long-term prognosis has not yet been determined, the mortality rate is extremely high, and most patients die during childhood [1]. The major characteristic of this disease is hypoplasia of organs due to disturbances in cell growth. Although the precise mechanism of this disease has not yet been clarified, endosomal dysfunction was speculated to be a possible mechanism for the cell proliferation defects observed in this disease [1].

To date, renal complications have been reported in seven patients with this disease [2–8]. Focal segmental glomerulosclerosis (FSGS) was diagnosed in two patients [2, 3], renal tubular acidosis in one [4], interstitial nephritis in one [4], renal hypoplasia in one [5], C1q nephropathy after bone marrow transplantation (BMT) in one [6, 7], and renal injury after BMT in one [8]. These lines of evidence indicate the importance of renal complications, although detailed clinical descriptions are lacking. Here, we report a girl with molecularly-confirmed MIRAGE syndrome with a particular emphasis on the detailed clinical information of her steroid-resistant nephrotic syndrome (SRNS). Consent for publication was obtained from her family.

## Case presentation

**Fig. 1:**
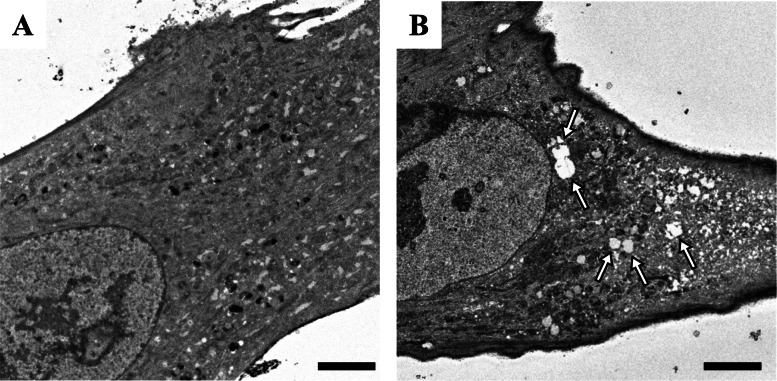
Ultrastructural findings of skin fibroblasts. Electron microscopic images (magnification, × 1,000) of biopsied skin fibroblast cells derived from a healthy child (**a**) and the MIRAGE syndrome patient with the p.L1539I *SAMD9* mutation (**b**). Giant vesicles without a specific inner structure were frequently observed (arrows). Bars, 5 μm

This case was a female born at 33 weeks gestational age with a birth weight of 1064 g, no asphyxia, and no family history. At birth, she showed transient low platelet counts (28,000/mm^3^), although it improved spontaneously. She had mild global developmental delays (rolling over, 8 months; walking with assistance, 16 months; standing alone, 19 months; walking without assistance, 27 months of corrected age). Her postnatal growth was poor. She had esophageal dysmotility, and she required tube-feeding for disturbances in oral intake. She was also suffering from clumpy abdominal pain and watery diarrhea. She had an episode of “autoimmune encephalitis” at age four. She developed recurrent aspiration pneumonia, and a gastrostomy was performed at age five. Whole exome sequencing was performed at age four, and a novel *SAMD9* variant (c.4615 T > A, p.Leu1539Ile) was identified. Biopsied skin fibroblast cells showed changes in the endosome system that are characteristic of MIRAGE syndrome, supporting the genetic diagnosis (Fig. 1). She did not show monosomy 7 nor myelodysplastic syndrome.

She showed recurrent infections (I), growth restriction (R), and intractable enteropathy (esophageal dysmotility, episodic vomiting, clumpy abdominal pain, and watery diarrhea) (E), although she did not suffer from myelosuppression (M) except for the transient low platelet count during the neonatal period. She also did not suffer from episodes of adrenal insufficiency (A) (basic cortisol level, 6.7 μg/dL; basic adrenocorticotropic hormone (ACTH) level, 12.8 pg/mL; peak cortisol level after corticotropin-releasing hormone (CRH) stimulation test, 13.2 μg/dL; peak ACTH level after CRH stimulation test, 58.7 pg/mL). She showed normal female external genitalia and a normal uterus (G). Her ovaries could not be detected by abdominal ultrasonography, which is not specific for her age. She showed insensitivity to pain with anhidrosis. The somatosensory-evoked potentials showed poorly depicted waveforms, and a sympathetic skin response was absent. These examinations indicated autonomic nerves dysfunction and peripheral neuropathy.

**Fig. 2:**
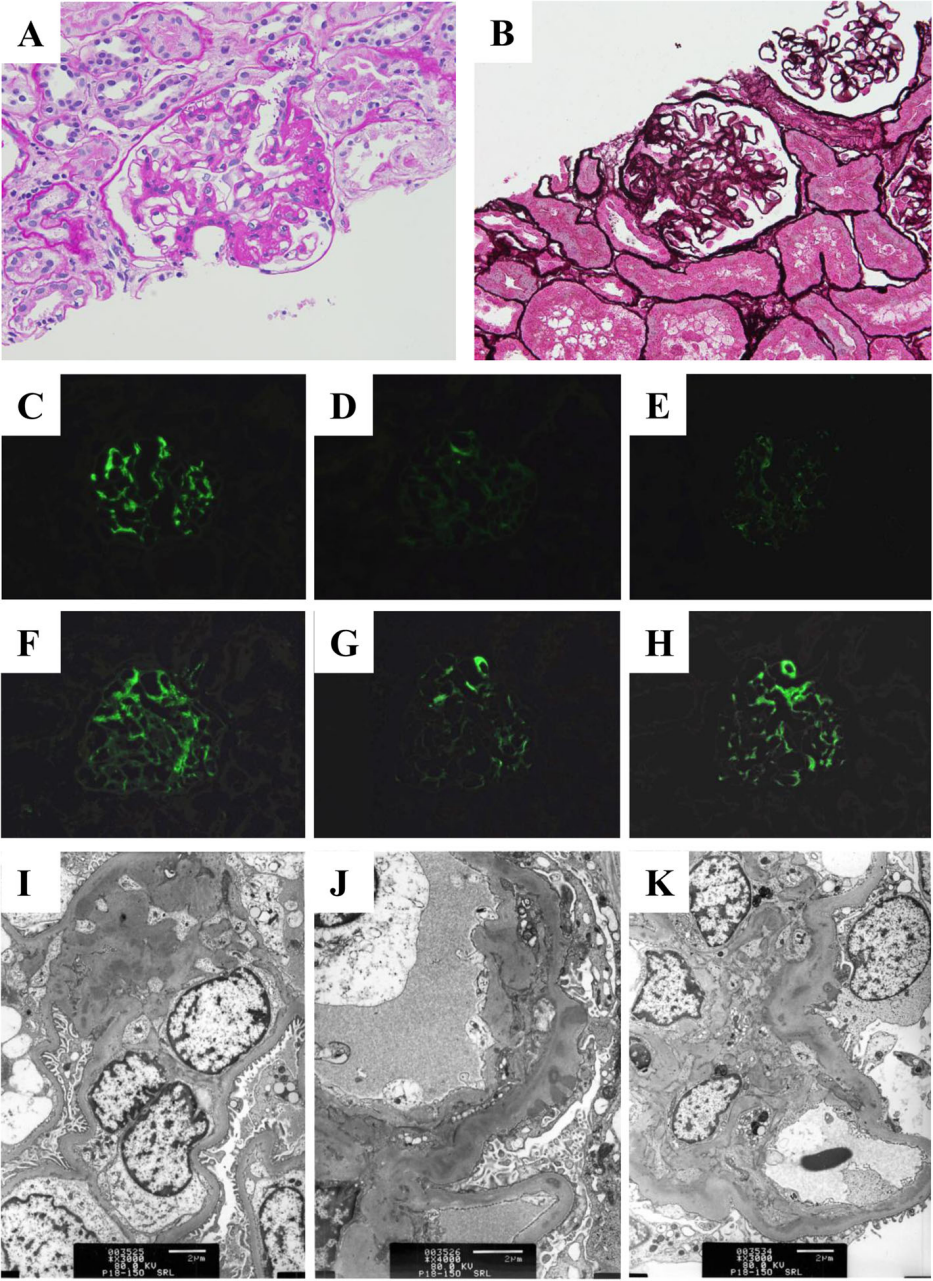
Kidney biopsy findings. **a**, **b**: Light microscopy shows FSGS (A, periodic acid-Schiff stain, × 200; B, periodic acid methenamine silver stain, × 200). **c**-**h**: Immunofluorescent microscopy (**c**, IgG; **d**, IgA; **e**, IgM; **f**, C3; **g**, C4; **h**, C1q). IgG (+), IgA (±), IgM (±), C3 (+), C4 (±), C1q (+). **i**-**k**: Electron microscopy (**i**, mesangial deposits; **j**, subepithelial deposits; **k**, subendothelial deposits)

Proteinuria was first noted at age one. Although heavy proteinuria persisted, her serum albumin levels remained normal until age three. Her serum albumin levels decreased to less than 3.0 g/dL at 3 years and 11 months, showing nephrotic syndrome. At this time, she was observed by outpatient clinic as she showed no edema. She developed prominent edema due to severe hypoalbuminemia of less than 2.0 g/dL at 5 years and 7 months. Renal biopsy revealed collapse in one glomerulus, segmental sclerosis in two glomeruli, and mild mesangial cell proliferation in 3 of 21 glomeruli (Fig. 2). Immunostaining showed codominant deposition of IgG, C3, and C1q in the mesangial area. Electron dense deposits were observed in mesangial matrix, subepithelial, and subendothelial space in electron microscopy. Mesangial interposition was also revealed. Mesangial matrix was slightly enlarged, although mesangial cells were not proliferated. Hyalinosis is found in some glomerular loops. Abnormal findings of epithelial cells were not detected except for partial disappearance of the foot process. The pathological diagnosis was FSGS. She received steroid treatment (60 mg/m^2^/day of oral prednisolone), although it did not lead to remission at 4 weeks. She was diagnosed with SRNS. Her treatment was switched from oral prednisolone to an angiotensin converting enzyme inhibitor (enalapril).

A physical examination at age six showed no abnormalities, except for extreme growth failure (height, 93.3 cm, − 4.4 SD; body weight, 10.0 kg, − 3.2 SD). Her white blood cell count (8070/μL), hemoglobin level (10.9 g/dL), and platelet count (419,000/μL) were within normal ranges. She displayed hypoalbuminemia (2.0 g/dL) with normal renal function (blood urea nitrogen level of 13.3 mg/dL and serum creatinine level of 0.26 mg/dL). Her serum immunoglobulin G level was low (228 mg/dL). Her complement activity, antinuclear antibody, anti-neutrophil cytoplasmic antibody, and anti-glomerular basement membrane antibody levels were normal. The urinalysis demonstrated heavy proteinuria (5 g/g⋅Cr) without hematuria. Her serum sodium (138 mEq/L), serum potassium (4.0 mEq/L), serum calcium (9.6 mg/dL), and serum phosphate (4.9 mg/dL) levels were normal, and the parameters of renal tubular functions were within normal ranges (fraction excretion of sodium, 0.2%; fraction excretion of potassium, 5.7%; calcium/creatinine ratio, 0.09; tubular reabsorption of phosphate, 96.2%; urinary β2 microglobulin, 0.32 μg/L), in which no sign of Fanconi syndrome was observed. The immunological examinations reported no abnormalities in the T cells and B cells, indicating normal cellular and humoral immunity. According to the abdominal ultrasound, her kidney size was normal for her body size (right kidney, 62 × 35 mm; left kidney, 61 × 30 mm).

Despite her heavy proteinuria, we decided not to initiate further immunosuppressive treatment such as cyclosporin or steroid-pulse therapy because we thought her nephrotic syndrome was genetic FSGS due to MIRAGE syndrome. At the last observation when she was 8 years old, her renal function was normal (serum creatinine level of 0.34 mg/dL and estimated glomerular filtration rate of 104 mL/min/1.73 m^2^) with mildly decreased serum albumin levels (2.9 g/dL) and persisting proteinuria (5.4 g/g Cr) under enalapril treatment.

## Discussion and conclusions

This case report describes a girl with MIRAGE syndrome who developed SRNS. A renal biopsy showed FSGS with immune deposits. This is the first detailed report of a MIRAGE syndrome patient with nephrotic syndrome.

Previously, there have been two case reports of FSGS with MIRAGE syndrome [2, 3]. We speculate that dysfunction of endocytosis in podocytes might play a possible role in proteinuria, leading to FSGS, although this was not demonstrated at the molecular level. Patients with MIRAGE syndrome have structural alterations in the endosomal system, including enlarged endosomes and the presence of structureless giant vesicles [1]. These findings were also observed in our patient. In recent years, animal studies have shown that endocytosis plays an important role in podocytes, and genetic defects involving the endosome system cause protein leakage [9–11]. Lysosomes are involved in processing endocytosed albumin in podocytes, and lysosomal dysfunction may contribute to podocyte injury and glomerulosclerosis [10].

A previous case report described a patient with renal tubular acidosis and a patient with interstitial nephritis [4]. Because immune abnormality is one of the major symptoms of MIRAGE syndrome, interstitial nephritis might be caused by autoimmune mechanisms of this disease. SAMD9 is shown to be expressed not only in glomeruli but also in renal tubules [12]. The proximal tubule plays a role in endocytosis via megalin or cubilin. However, the patient’s renal tubular function was normal, and she did not suffer from Fanconi syndrome. Furthermore, the renal biopsy did not show interstitial nephritis.

Because this patient showed the codominant deposition of IgG, C3, and C1q on the glomeruli, nephrotic syndrome might have resulted from the immune abnormalities of this syndrome. C1q nephropathy was previously reported in a patient with MIRAGE syndrome [6, 7], although this case developed nephrosis after BMT. C1q deposition usually indicates immune-complex mediated glomerulopathy and is typically observed in systemic lupus erythematosus. However, the clinical significance of C1q nephropathy remains obscure [13]. Light microscopic features of C1q nephropathy are variable and heterogeneous, such as minor glomerular abnormalities, FSGS, and sometimes membranous nephropathy [14]. Its deposition is sometimes non-specifically observed. There has even been a case report of a girl with congenital nephrotic syndrome with granular mesangial C1q deposition on her glomeruli, although congenital nephrotic syndrome is typically a genetic disease [15].

Hyperfiltration of glomeruli due to reduced nephron by premature birth might be one of the mechanisms of proteinuria as she was born at 33 weeks gestational age with a birth weight of 1064 g. However, nephrotic range proteinuria is relatively rare on this situation. Moreover, her glomeruli were not large, which was not typical for FSGS due to hyperfiltration by nephron reduction.

The patient’s kidney size was normal for her body weight, and her renal function was normal. Although the major characteristics of this disease include hypoplasia of organs due to disturbances of cell growth and renal hypoplasia is also reported in a patient with this disease [5], hypoplastic kidney was not observed in this case.

Other specific symptoms of this case were insensitivity and anhidrosis. To date, three cases of hypolacrima with corneal ulcer [4, 16, 17], one case of anhidrosis [8] and two cases of hyperhidrosis [4, 17] have been reported in MIRAGE syndrome. There have been no reports of insensitivity. We believe dysautonomia, such as insensitivity and anhidrosis, might be important clinical symptoms of this syndrome. More clinical information should be accumulated in the future.

We decided not to introduce immunosuppressive agents. We only continued the angiotensin converting enzyme inhibitor as the conservative treatment because we thought this case was genetic FSGS, although recent review article did not introduce “SAMD9” as a causative gene of SRNS [18]. Until now, her renal function has remained normal, although proteinuria persisted under enalapril treatment for three years after the onset of nephrotic syndrome. The long-term prognosis of renal function is obscure in this disease; thus, it is necessary for us to follow-up and observe the renal function of this patient for a long time. We also have to obtain more information about the renal complications of this disease by evaluating more cases to determine the mechanism of this rare disease in the future.

## Data Availability

The datasets used and/or analyzed during the current study are available from the corresponding author upon reasonable request.

## Abbreviations

FSGS: Focal segmental glomerulosclerosis
BMT: Bone marrow transplantation
ACTH: Adrenocorticotropic hormone
CRH: Corticotropin-releasing hormone
SRNS: Steroid-resistant nephrotic syndrome
